# Perioperative hypothermia and incidence of surgical wound infection: a bibliographic study

**DOI:** 10.1590/S1679-45082014RW2398

**Published:** 2014

**Authors:** Aline Batista da Silva, Aparecida de Cassia Giani Peniche

**Affiliations:** 1 Universidade de São Paulo, São Paulo, SP, Brazil.; 2 Escola de Enfermagem, Universidade de São Paulo, São Paulo, SP, Brazil.

**Keywords:** Hypothermia, Perioperative care, Surgical wound infection

## Abstract

The purpose of this review article was to understand and analyze the scientific production related to the occurrence of perioperative hypothermia and the incidence of infection on the surgical site. For this purpose, a search was conducted in the databases LILACS, MEDLINE, PubMed, CINAHL and Cochrane, using the health science descriptors DECS, from 2004 to 2009. A total of 91 articles were found. After eliminating duplicate items and using selection criteria for inclusion, six manuscripts remained for analysis. The studies were classified as retrospective, prospective, case studies, and clinical trials. After analysis, the majority of studies showed that hypothermia must be prevented during the perioperative period to reduce complications in the healing process of the surgical incision. Therefore, unadverted hypothermia directly influences in surgical site healing, increasing the incidence of infection in the surgical wound.

## INTRODUCTION

Vital signs are health indicators that demonstrate the efficacy of body functioning, be it circulatory, respiratory, neural or endocrine. Changes in vital signs often times indicate the need for medical or nursing interventions to restore normal pattern. Body temperature is an extremely important sign for surgical patients.^(1)^

Anesthesia and surgery encompass many risks in regard to maintaining the normal pattern of vital signs, as well as of temperature. Hypothermia is a common perioperative phenomenon that may cause relevant complications for surgical patients.

Body temperature control is attained by the balance between heat production and heat loss. Production is carried out by factors determining the body metabolic rate, such as baseline metabolism of body cells, and extra metabolism from muscle activities, hormone action, and others. Heat loss occurs in two ways: conduction of deep tissue to the skin, and transference of skin heat to the environment. This transference occurs by means of four phenomena: radiation (infrared rays irradiating from the body); conduction (heat transfer directly from the body surface to solid objects); convection (occurs after conduction of body heat to air, by air currents which surround the body), and evaporation (due to sweat evaporation).^(2)^

Thermoregulation is guaranteed by the action of the hypothalamus, which, by neural feedbacks, maintains body temperature within normal ranges (36.1 to 37.8°C), thanks to the phenomena already described.^(3)^

Hypothermia is defined as central temperature ≤36°C, and it may be classified in mild (34 to 36°C), moderate (30 to 34°C) and severe (<30°C). Perioperative hypothermia often occurs, and it may be intentional (for vital organ protection) or non-intentional. Nevertheless, it is neither valued nor treated as it should, especially in relation to its implications, which may be extremely deleterious to surgical patients.^(3)^

The operating room is an environment prone to the development of hypothermia, since it associates: the cold environment of the operating room, the skin antisepsis with the body uncovered, the infusion of cold solutions during the procedure, and the use of anesthetic drugs that change the thermoregulation mechanism.

Among the complications from perioperative hypothermia we find increased wound infection rate, since hypothermia increases sensitivity to infections in this type of wound, due to vasoconstriction and immunity impairment.

Surgical incision suffers relevant changes when there is a non-intentional uncontrolled decrease in body temperature, which acts directly over antibodies and immune defense cells, and indirectly decreasing tissue oxygen, due to vasoconstriction.^(4-18)^

To avoid postoperative complications due to hypothermia, there are practices to prevent this phenomenon, which utilize active warming practices for the perioperative protection of the patient, aiming to reducethe adverse effects of this phenomenon.^(5-12)^

The objective of this study as to find, by means of literature review, scientific evidence of the correlation between perioperative hypothermia and increased rate of wound infection, since hypothermia is a recurrent phenomenon in the surgical environment.

## METHODS

It is a literature review, expanded from scientific initiation conducted in 2009/2010, by the *Escola de Enfermagem da Universidade de São Paulo* (USP)^(19)^ that tried to answer the following question: is there a correlation between the occurrence of perioperative hypothermia and the increased rate of surgical wound infection?

The study period was from 2004 to 2011 and encompassed the publications identified in the following database searches: LILACS, MEDLINE, PubMed, CINAHL, and Cochrane.

For data collection, terms were identified in the *Descritores em Ciências da Saúde *(DECS) (Health Sciences Descriptors). The PubMed methodology was adopted, known as PICO, P standing for patient, I for intervention, C for comparison group, and O for outcome. The search was then structured as flows: P (surgery), I (hypothermia), O (surgical wound dehiscence OR surgical wound infection OR infection).

The following inclusion criteria were defined: scientific publications related to perioperative hypothermia and increased incidence in surgical wound for 19-year-old adults or older. Exclusion criteria were: experimental studies with animal models, in children, and those with non-surgical patients or that approached other causes for infection of the surgical wound, protocols, systematic or literature reviews, and pathophysiologic studies.

The articles were divided according to the following categorization: case studies, retrospective studies, prospective studies, and clinical trials.

A total of 55 articles were found in PubMed, 29 in MEDLINE, 14 in CINAHL, 3 in LILACS, and 2 in Cochrane. Ninety-one references were found, but only 6 articles met the inclusion criteria. Duplicated articles and those where the abstract did not answer the core question of the study were eliminated.

## RESULTS

The distribution of the articles according to their categorization is depicted on table 1.

**Table 1:** Distribution of the articles according to the category 2004/2011

Type	Articles (n)
Case studies	2
Retrospective studies	2
Clinical trials	1
Prospective study	1

### Case studies

**Figure 1:**
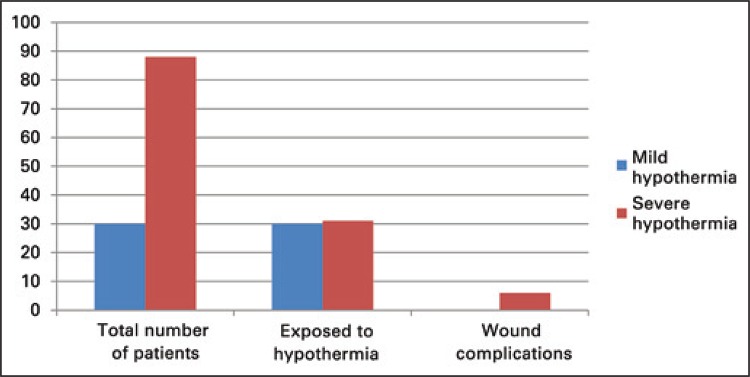
Relation between mild hypothermia and severe hypothermia and surgical wound infection

Studies about specific procedures using hypothermia. Controlling hypothermia was decisive to heal the surgical wound. As an example, the induction of mild hypothermia during an emergency procedure in a kidney transplant recipient for total aortic arch replacement; there were no side effects of hypothermia, and the healing process of the surgical wound occurred normally.^(20)^ Another study reported Stanford type A operations in 88 patients, 31 being induced to profound hypothermia; 6 had delayed healing of the surgical wound and local infection (Figure 1).^(21)^

### Retrospective studies

There were two retrospective studies that reported important data on perioperative hypothermia.^(22,23)^

**Figure 2:**
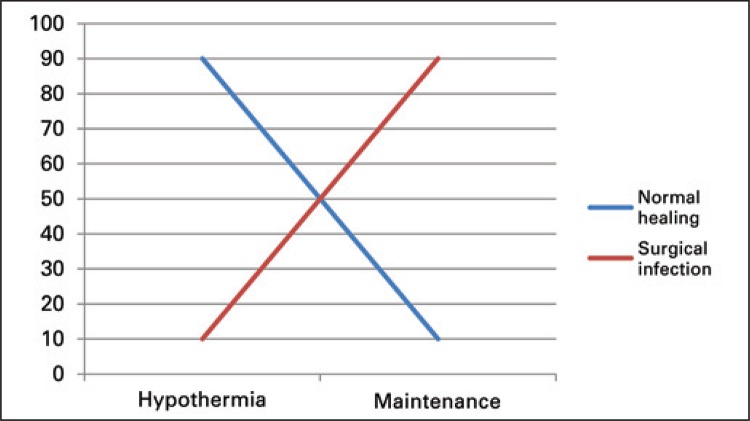
Correlation between surgical wound infection in the presence of hypothermia and temperature maintenance

An analysis conducted in 1,446 patients submitted to bowel surgery observed that those with slightly lower temperature (35.8±0.8°C *versus* 36.0±0.9°C) had a lower risk for surgical wound infection.^(22) ^This result stood out since it demonstrated a correlation opposite to the one expected (Figure 2).

**Figure 3:**
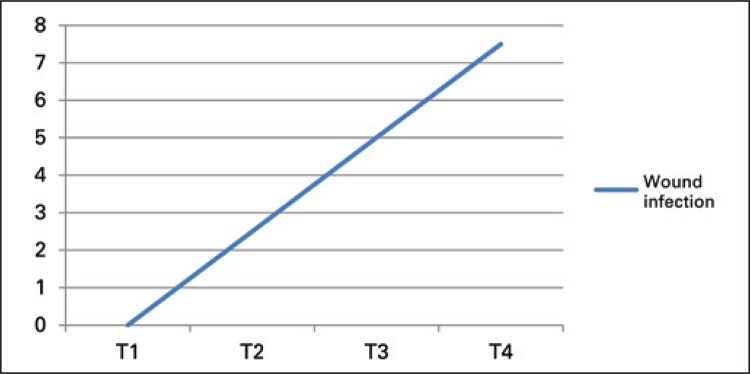
Correlation between exposure time to hypothermia and incidence of surgical wound infection

In another investigation, 70 patients submitted to spinal surgery with intentional hypothermia were examined. The patients exposed to brief periods of hypothermia did not present adverse effects, whereas those who stayed prolonged periods in hypothermia presented higher risk for surgical wound infection (Figure 3).^(23)^

### Clinical trial

The purpose of one of the studies was to analyze the cell immunity mechanisms affected by perioperative hypothermia and that influence the healing process of the surgical site.^(24)^ Samples of healthy volunteers exposed to different temperatures for 4 hours were analyzed. The results were: reduction in HLA-DR expression in surface monocytes, resulting in innate immune response with greater phagocytosis and activation of the adaptive immune system; delay in tumor necrosis factor alpha (TNF-α) clearance, what may trigger tissue lesion; increased release of interleukin 10 (IL-10); and, as a consequence, exacerbated inflammatory response.

### Prospective study

The objective of one of these studies was to analyze the effect of fluid heating in prevention of hypothermia in myocardial revascularization. The study was randomized and encompassed 40 patients, 20 in the control group, and 20 in the experimental group. The control group was submitted to methods for the prevention of hypothermia regularly used in the organization (operating room temperature raised to 25°C and use of warm water mattress at 38°C), while the experimental group was submitted to the same practices plus fluids warmed to 41°C.^(25)^ The temperature of the experimental group reduced gradually but there were no significant systemic differences between the two groups. The conclusion was that fluid warming contributed to prevent decrease in systemic temperature during myocardial bypass surgery, acting in a preventive way in complications derived from perioperative hypothermia and being included in the prevention of surgical wound infection.

## DISCUSSION

The data presented in the literature demonstrate that hypothermia is recurrent in anesthetic surgical procedures, neither being valued, nor treated as it should. Scientific evidences point to the use of active measures for the prevention of unintentional hypothermia, thus reducing the risk for surgical wound infection. It is important to monitor the temperature of the surgical patient during the whole perioperative period, besides offering warming methods from the transport to the operating room, during stay in the operating room, until anesthetic recovery and discharge. The infusion of warm fluids also plays important role in maintaining body temperature during that process.^(5-9)^

There is scientific proof of the benefits of the use of warming by forced air in maintaining normothermia.^(14,21)^

The healing process is directly affected by the occurrence of perioperative hypothermia, since immune defense cells are changed by the temperature decrease, and tissue oxygen supply is reduced due to hypothermic vasoconstriction.^(16,24)^

The patient temperature must be a relevant part of vital signs which needs to be more valued during the anesthetic-surgical procedure, since its variation may be extremely harmful to the patient. The multiprofessional team must be aware of prevention of hypothermia and its complications. Its occurrence may bring many problems to the surgical patient, increase length of stay, and increase costs.

Analyzing the data presented, there is no clear evidence about the occurrence of perioperative hypothermia and surgical wound infection, but there is evidence indicatingthat maintaining normothermia in surgical patients influences in comfort and decreases the risk for these patients. It is the duty of the operating room nurse to take ownership of scientific basis which guarantee care protocols for the prevention of perioperative hypothermia and therefore act in a safe way in the care of the patient who is in an environment full of risks.

## CONCLUSION

Perioperative hypothermia is a phenomenon that happens often, not valued and/or treated as it should. Its manifestation during the anesthetic-surgical process is directly connected to the many disturbances the patients go through, including the occurrence of surgical wound infection.

It is important to emphasize that perioperative hypothermia is preventable, the surgical nurse having the important role of acting for the sake of patient safety, avoiding the occurrence of this event during the perioperative period. The nurse’s actions must go from when the patient is transported from the ward until he/she is back to it, using active methods for warming and body surface protection against heat loss.
