# Supplementary material for: Perioperative hypothermia and incidence of surgical wound infection: a bibliographic study
Source: Einstein (Sao Paulo). 2014 Oct-Dec;12(4):513–7. doi: 10.1590/S1679-45082014RW2398 (PMC4879923; doi:10.1590/S1679-45082014RW2398)
Supplement: Translation

# Translation: Hipotermia perioperatória e aumento de infecção da ferida cirúrgica: estudo bibliográfico

**Authors:** Aline Batista da Silva, Aparecida de Cassia Giani Peniche

**Affiliations:** 1 Universidade de São Paulo, São Paulo, SP, Brasil.; 2 Escola de Enfermagem, Universidade de São Paulo, São Paulo, SP, Brasil.

**Keywords:** Hipotermia, Assistência perioperatória, Infecção da ferida operatória

## Abstract

A proposta deste artigo foi conhecer e analisar a produção científica relacionada à ocorrência de hipotermia perioperatória e à incidência de infecção no sítio cirúrgico. Foi realizado um levantamento nas bases de dados LILACS, MEDLINE, PubMed, CINAHL e Cochrane, com descritores do DECS, em um período de 2004 a 2009. Foram encontrados 91 artigos. Após a eliminação dos artigos duplicados e da seleção por critérios de inclusão, restaram seis manuscritos para análise. Os estudos foram divididos em análise retrospectiva, estudos de caso, ensaio clínico e estudo prospectivo. Após a análise dos estudos, a maioria apontou que a hipotermia deve ser prevenida durante o perioperatório, a fim de diminuir as complicações na cicatrização da incisão cirúrgica. Assim, a hipotermia inadvertida influi diretamente no processo de cicatrização do sítio cirúrgico, aumentando a incidência de infecção da ferida operatória.

## INTRODUÇÃO

Os sinais vitais são indicadores de saúde que demonstram a eficácia da função corporal, seja circulatória, respiratória, neural ou endócrina. A alteração dos sinais vitais, muitas vezes, indica a necessidade de intervenções médicas ou de enfermagem, para restaurar o padrão de normalidade. A temperatura corpórea é um sinal de grande importância para o paciente cirúrgico.^(1)^

O procedimento anestésico-cirúrgico envolve inúmeros riscos a manutenção do padrão de normalidade dos sinais vitais, bem como da temperatura, sendo a hipotermia um fenômeno comum no perioperatório e que pode acarretar complicações relevantes ao paciente cirúrgico.

O controle da temperatura corporal é obtido por meio do equilíbrio existente entre a produção e a perda de calor. A produção é realizada por fatores que determinam a taxa metabólica do organismo, como o metabolismo basal das células do corpo, e o metabolismo extra por meio de atividades musculares, por ação hormonal e outras. A perda de calor ocorre por dois aspectos: pela condução dos tecidos profundos até a pele e pela transferência do calor da pele para o ambiente. Essa transferência se dá por meio de quatro fenômenos: irradiação (raios infravermelhos que se irradiam do corpo); condução (transferência de calor direto da superfície corporal para objetos sólidos); convecção (ocorre após a condução do calor corporal para o ar, por meio de correntes de ar ao redor do corpo) e evaporação (pela evaporação do suor corpóreo).^(2)^

A termorregulação é garantida pela ação do hipotálamo, que, por mecanismos de *feedback* neurais, consegue manter a temperatura corporal normal (36,1 a 37,8°C), dados os fenômenos já aqui descritos.^(3)^

A hipotermia é definida como temperatura central do organismo ≤36°C, podendo ainda ser classificada como leve (34 a 36°C), moderada (30 a 34°C) e grave (<30°C). A hipotermia perioperatória ocorre frequentemente, podendo ser intencional (com a finalidade de proteger órgãos vitais) ou não intencional. Porém, ela não é valorizada e tratada como deveria ser, principalmente quando relacionada às suas implicações, que podem ser extremamente prejudiciais ao paciente cirúrgico.^(3)^

O centro cirúrgico é um ambiente propício para o desenvolvimento da hipotermia, pois associa: o ambiente frio da sala de operação, a realização da antissepsia da pele do paciente com o corpo descoberto, a infusão de soluções frias no decorrer do procedimento e o uso de drogas anestésicas que alteram o mecanismo da termorregulação.

Dentre as complicações resultantes da hipotermia no período perioperatório, está o aumento do índice de infecção da ferida operatória, pois a ocorrência da hipotermia aumenta a suscetibilidade a infecções desse tipo de ferida, em razão da vasoconstrição e do comprometimento da imunidade.

A incisão cirúrgica sofre alterações relevantes quando ocorre a diminuição não intencional e não controlada da temperatura corpórea, a qual acontece de forma direta pela atuação em anticorpos e células de defesa imunitária, e de forma indireta pela diminuição na oxigenação tecidual, devido à vasoconstrição.^(4-18)^

Para evitar complicações pós-cirúrgicas advindas da hipotermia, existem práticas para a prevenção desse fenômeno, as quais se utilizam de medidas ativas de aquecimento para a proteção do paciente no perioperatório, visando reduzir os efeitos adversos desse fenômeno.^(5-12)^

Este estudo teve por objetivo buscar, por meio de revisão de literatura, evidências científicas da relação entre hipotermia no perioperatório e o aumento da incidência de infecção da ferida operatória, já que a hipotermia é um fenômeno recorrente no ambiente cirúrgico.

## MÉTODOS

Trata-se de uma revisão de literatura, ampliada a partir de iniciação científica realizada em 2009/2010 pela Escola de Enfermagem da Universidade de São Paulo (USP),^(19)^ que buscou responder a seguinte questão: existe relação entre a ocorrência de hipotermia perioperatória e o aumento da infecção da ferida operatória?

O período de estudo foi de 2004 a 2011 e envolveu os trabalhos identificados na busca eletrônica nas seguintes bases de dados: LILACS, MEDLINE, PubMed, CINAHL e Cochrane.

Para a coleta de dados, foram identificados termos no Descritores em Ciências da Saúde (DECS). Foi adotada a metodologia do PUBMED, conhecida por PICO, sendo P (*patient) *correspondente a “paciente”, I (*intervention*) à “intervenção”, C (*comparison group)* a “grupo de comparação” e O (*outcome)* a “resultado”. A busca ficou então estruturada da seguinte forma: P (*surgery*), I (*hypothermia*), O (*surgical wound dehiscence* OR *surgical wound infection* OR *infection*).

Foram definidos os seguintes critérios de inclusão: artigos científicos relacionados à ocorrência de hipotermia no perioperatório e o aumento da incidência de infecção da ferida operatória em adultos acima de 19 anos. Foram critérios para exclusão: estudos experimentais com modelos animais, em crianças e aqueles envolvendo pacientes não cirúrgicos ou que abordassem outras causas de infecção da ferida operatória, protocolos, revisões sistemáticas ou bibliográficas, e estudos fisiopatológicos.

Os artigos foram dividos segundo a seguinte categorização: estudos de caso, estudos retrospectivos, estudo prospectivo e ensaio clínico.

Foram encontrados 55 artigos no PubMed, 29 no MEDLINE, 14 na CINAHL, 3 no LILACS e 2 na Cochrane. No total, foram localizadas 91 referências bibliográficas, mas somente 6 artigos atenderam aos critérios de inclusão. Foram eliminados artigos duplicados e aqueles cujos resumos não respondiam a questão norteadora do estudo.

## RESULTADOS

Apresenta-se a distribuição dos artigos de acordo com sua categorização na tabela 1.

**Tabela 1:** Distribuição dos artigos de acordo com a categoria 2004/2011

Enfoque	Artigos (n)
Estudos de caso	2
Estudos retrospectivos	2
Ensaio clínico	1
Estudo prospectivo	1

### Estudos de caso

Foram apresentados estudos em procedimentos específicos nos quais a hipotermia esteve presente. Seu controle foi decisivo para a cicatrização da ferida operatória. Como, por exemplo, a indução à hipotermia leve, durante uma cirurgia de substituição de emergência total do arco aórtico para um receptor de transplante renal; constatou-se que não houve efeitos colaterais dessa hipotermia, sendo que o processo cicatricial da incisão cirúrgica ocorreu normalmente.^(20)^ Outro estudo relatou operações do tipo A de Stanford em 88 pacientes, sendo 31 pacientes induzidos à hipotermia profunda; 6 deles sofreram atraso na cicatrização da ferida operatória e tiveram infecção local (Figura 1).^(21)^

**Figura 1:** Relação entre hipotermia leve e hipotermia profunda e a infecção da ferida operatória

### Estudos retrospectivos

Foram apresentados dois estudos retrospectivos que relatavam dados importantes sobre a hipotermia perioperatória.^(22,23)^

Análise realizada em 1.446 pacientes submetidos à cirurgia de intestino constatou que aqueles com temperatura levemente mais baixa (35,8±0,8°C *versus* 36,0±0,9°C) tiveram um menor risco de infecção da ferida operatória.^(22) ^Esse resultado chamou a atenção por mostrar uma relação inversa à esperada (Figura 2).

**Figura 2:** Relação entre a infecção da ferida cirúrgica na ocorrência de hipotermia e a manutenção da temperatura

Em outra pesquisa, foram analisados 70 pacientes submetidos à cirurgia espinhal, sendo a hipotermia intencional. Os pacientes que ficaram expostos a breves períodos de hipotermia não apresentaram efeitos adversos; já os que ficaram em períodos prolongados de hipotermia apresentaram mais riscos de infecção da ferida operatória (Figura 3).^(23)^

**Figura 3:** Relação entre o tempo de exposição à hipotermia e o aumento de infecção na ferida cirúrgica

### Ensaio clínico

O intuito de um dos estudos foi analisar os mecanismos imunológicos celulares afetados pela hipotermia perioperatória e que influi no processo de cicatrização do sítio cirúrgico.^(24)^ Foram analisadas amostras de voluntários saudáveis expostas por 4 horas a diferentes temperaturas. Os resultados foram: redução na expressão de HLA-DR em monócitos de superfície, o que resultou em resposta imune inata com maior fagocitose e ativação do sistema imune adaptativo; atraso no *clearance* de fator de necrose tumoral alfa (TNF-α), o que pode desencadear a lesão tecidual; aumento da liberação de interleucina 10 (IL-10); e, consequentemente, uma resposta inflamatória exacerbada.

### Estudo prospectivo

O objetivo de um dos estudos foi analisar o efeito do aquecimento de fluídos na prevenção da hipotermia em cirurgias de revascularização miocárdica. O estudo foi randomizado e apresentou 40 pacientes, sendo 20 do grupo controle e 20 do grupo experimental. O grupo controle foi submetido a métodos de prevenção da hipotermia aplicados na instituição (aumento da temperatura da sala de operação em 25°C e uso de colchão de água aquecida 38°C), enquanto o grupo experimental foi submetido às mesmas práticas e também a fluidos aquecidos em 41°C.^(25) ^A temperatura do grupo experimental reduziu gradativamente, porém não houve diferenças sistêmicas significantes entre os grupos. Concluiu-se que o aquecimento de fluidos contribui para impedir a diminuição da temperatura sistêmica durante a cirurgia de revascularização do miocárdio, atuando de forma preventiva nas complicações decorrentes de hipotermia perioperatória e sendo incluído na prevenção da infecção da ferida cirúrgica.

## DISCUSSÃO

Os dados apresentados na literatura mostram que a hipotermia é recorrente no procedimento anestésico cirúrgico, não sendo valorizada e nem tratada como deveria. As evidências científicas apontam para utilização de medidas ativas para a prevenção da hipotermia inadvertida, reduzindo, assim, os riscos de infecção da ferida operatória. É importante monitorizar a temperatura do paciente cirúrgico em todo o período perioperatório, além de oferecer métodos de aquecimento, desde o transporte dele ao centro cirúrgico, durante sua estadia na sala de operação, até o momento da recuperação anestésica e alta. A infusão de fluídos aquecidos também tem papel importante na manutenção da temperatura corpórea durante esse processo.^(5-9)^

Há comprovação científica dos benefícios da utilização do aquecimento por meio de ar forçado na manutenção da normotermia.^(14,21)^

O processo cicatricial é diretamente afetado pela ocorrência de hipotermia perioperatória, pois as células de defesa imunitária são alteradas pela diminuição de temperatura, e a oferta de oxigênio tecidual é reduzida devido à vasoconstrição hipotérmica.^(16,24)^

A temperatura do paciente deve ser um dado relevante de sinal vital, que necessita ser mais valorizado durante o procedimento anestésico-cirúrgico, já que a variação desta pode acarretar sérios danos ao paciente. A equipe multiprofissional deve estar atenta para a prevenção da hipotermia e suas complicações. Sua ocorrência pode significar inúmeros danos ao paciente cirúrgico, aumentar o tempo de hospitalização e elevar os custos com o paciente.

Pela análise dos dados apresentados, não há clara evidência sobre a ocorrência da hipotermia no período perioperatório e a infecção da ferida cirúrgica, mas existem evidências que indicam que a manutenção da normotermia do paciente cirúrgico influencia no conforto e na diminuição dos riscos para esse paciente. Cabe ao enfermeiro de centro cirúrgico apropriar-se de bases científicas que garantam protocolos assistenciais para a prevenção da hipotermia perioperatória e, assim, atuar de forma segura na assistência ao paciente que se encontra em um ambiente repleto de riscos.

## CONCLUSÃO

A hipotermia perioperatória é um fenômeno que ocorre frequentemente, não sendo valorizada e/ou tratada como deveria ser. Sua manifestação, durante o processo anestésico-cirúrgico, está ligada diretamente aos inúmeros transtornos ao paciente, incluindo a ocorrência de infecção da ferida cirúrgica.

É importante salientar que a hipotermia perioperatória pode ser prevenida, cabendo ao enfermeiro de centro cirúrgico o importante papel de atuar pela segurança do paciente, evitando a ocorrência desse evento durante o período perioperatório. A atuação do enfermeiro deve ser ativa desde o transporte do paciente de sua unidade de internação até seu retorno, utilizando métodos ativos de aquecimento e proteção da superfície corpórea do paciente contra a perda de calor.
